# Identification and Expression Profiles of IL-8 in Bighead Carp (*Aristichthys nobilis*) in Response to Microcystin-LR

**DOI:** 10.1007/s00244-013-9910-8

**Published:** 2013-06-25

**Authors:** Huiying Li, Yan Cai, Ping Xie, Guangyu Li, Le Hao, Qian Xiong

**Affiliations:** 1 Donghu Experimental Station of Lake Ecosystems, State Key Laboratory of Freshwater Ecology and Biotechnology of China, Institute of Hydrobiology, Chinese Academy of Sciences, Wuhan, 430072 People’s Republic of China; 2 Present Address: Key Laboratory of Plant Germplasm Enhancement and Specialty Agriculture, Wuhan Botanical Garden, CAS, Lumo Street, Wuhan, 430074 People’s Republic of China

## Abstract

Microcystin-LR (MCLR) is a widespread cyanotoxin and has immunotoxicity to animals, including fish. Chemokines are considered to play important roles in inflammatory response induced by MCLR. In this study, we cloned the full-length cDNA of *interleukin*-*8* (*IL*-*8*) from bighead carp (*Aristichthys nobilis*) for the first time. The full-length *IL*-*8* cDNA was 552 bp and contained a 297-bp open-reading frame that encoded for a 98-amino acid protein. The deduced IL-8 protein had a typical aspartic acid (D)-leucine (L)-arginine (R) and a CXC motif at the N-terminal, which were conserved in most fish species. Phylogenetic analysis showed that bighead carp IL-8 protein was grouped in the teleost IL-8 lineage 2. Under normal conditions, the expression of *IL*-*8* is constitutive and weak in all tested tissues. However, MCLR treatment could significantly increase the transcription of *IL*-*8* in bighead carp in a temporal- and dose-dependent pattern. The present study will help us to understand more about the evolution of *IL*-*8* and its function in the MCLR induced proinflammatory response in bighead carp.

Chemokines are a family of small cytokines secreted by cells (Laing and Secombes 2004). The CXC chemokines are the principal group in the chemokine family, which can be subdivided into two categories, those with an glutamate (E)-leucine (L)-arginine (R) motif (ELR-positive) and those without the motif (ELR-negative) (Fernandez and Lolis 2002). Interleukin-8 (IL-8) is a member of the CXC chemokine family that can be produced by a wide variety of cells in response to many stimulants. IL-8 is synthesized as a precursor with a signal peptide, which is proteolytically processed to release an 8-kD molecule (Harun et al. 2008). The active form of IL-8 is a dimmer and is comprised of two identical subunits. IL-8 monomer contains a triple-strand antiparallel β-sheet, whereas the dimer comprises a six-strand antiparallel β-pleated sheet and is spanned by two long symmetry-related α-helices, which locate in the carboxy terminal of the two subunits (Clore et al. 1990).

The primary function of IL-8 is the recruitment of monocytes and neutrophils, the signature cells of acute inflammatory response (Remick 2005). Inflammatory cells follow the increasing chemokine concentration and move toward the source of the chemokines. Another pivotal function of IL-8 is to activate monocytes and neutrophils. The biological effects of IL-8 are mediated through the binding of IL-8 to two cell surface receptors, CXCR1 and CXCR2 (Holmes et al. 1991). IL-8 is also an ideal molecule for locating at acute inflammatory sites. It is resistant to temperature, proteolysis, and some acidic environments and is relatively stable at acute inflammatory sites. However, IL-8 is highly sensitive to oxidants, and antioxidants can substantially decrease IL-8 gene expression (DeForge et al. 1993).

Microcystins (MCs) are produced by various species of toxic cyanobacteria and are known as potent hepatotoxins. MCs mainly affect the liver, causing minor to widespread damage depending on the amount of toxin absorbed (Butler et al. 2009). Evidence has indicated that MCs also have immunotoxicity to animals or culture cells (Mankiewicz et al. 2002; Shen et al. 2003). MCs can regulate the production of interleukin (IL)-1 and tumor-necrosis factor (TNF)-α, induce nitric oxide synthesis in macrophages (Pahan et al. 1998; Rocha et al. 2000), and modulate the spontaneous adherence of human peripheral polymorphonuclear leukocytes (PMNLs; Hernández et al. 2000). Moreover, it has been suggested that MCs downregulate lymphocyte functions through decreasing the mRNA stability of IL-2. In addition, MCs depress lymphocytes response to lipopolysaccharide and produce dose-dependent inhibition of polyclonal antibody response in vitro (Yea et al. 2001).

IL-8 was initially purified as a neutrophil chemoattractant in human MNLs (Yoshimura et al. 1987). Since then, many IL-8 sequences have been identified. Two IL-8 lineages have been identified in multiple teleost species (Abdelkhalek et al. 2009). Both lineages were likely to have arisen from a common ancestor of mammalian IL-8/ELR+CRC chemokines (Van der Aa et al. 2010). Lineage 1 is present in most fish, such as carp (Huising et al. 2003), zebrafish (Nomiyama et al. 2008), trout (Laing et al. 2002), haddock (Corripio-Miyar et al. 2007), fugu (Covello et al. 2009), flounder (Lee et al. 2001) and Atlantic cod. Lineage 2 is found only in cyprinids and zebrafish (Van der Aa et al. 2010). However, to date, there has been no report about IL-8 in bighead carp (*Aristichthys nobilis*).

Bighead carp is a commercially important fish in China. Its living habits suggest that it might consume great quantities of MC-producing cyanobacteria. As a freshwater phytoplanktivorous fish, bighead carp is more resistant to the toxic effects of MCLR compared with mammals (He et al. 1997; Xie et al. 2004). Therefore, bighead carp has received extensive research interest as a model species for toxicology, ecology, physiology, evolutionary genetics, and etc. (Xie 2003).

In our previous study, we obtained cDNA fragments of *IL*-*8* gene from bighead carp injected intraperitoneally with 200 μg MCLR/kg body weight (bw) using suppression subtractive hybridization (SSH) technique and found that it was upregulated in response to MCLR treatment (unpublished data). To better understand the evolution of *IL*-*8*, as well as its role in the anti-MCLR response in bighead carp, we cloned the full-length cDNA of *IL*-*8* using RACE method. Using semiquantitative real-time PCR (Q-PCR) assay, we confirmed that *IL*-*8* expression was induced in bighead carp liver by MCLR. The molecular characteristics of bighead carp *IL*-*8* and gene expression profiles were further analyzed.

## Materials and Methods

### Toxin

MCLR was isolated from surface cyanobacterial blooms (mainly *Microcystis aeruginosa*) collected from Lake Dianchi in China and then purified and quantified using an improved Ramanan method (Li et al. 2009). The product purity was >97 %. MCLR was dissolved in water.

### Bighead Carp Exposure Experiment and Sample

Healthy bighead carp without MCLR contamination, weighing 899 ± 251 g, were purchased from a local cauf (Wuhan China) and acclimated for 14 days before experimentation in a 150-L volume with 6 bighead carp in each group. Water temperature was kept at 25 ± 2 °C, pH 7.4 ± 0.9, dissolved oxygen value 6.8 ± 0.7 mg/L under a 12:12h light-to-dark cycle. The fish were fed with dry commercial feed at a rate of 2 % bw/d. No food was fed to bighead carp 2 days before and during the course of the experiment. All experimental research on bighead carp were performed with the approval of the Animal Ethics Committee at the Institute of Hydrobiology, Chinese Academy of Sciences (study ID no. Y11309-1-201).

According to the method of Li et al. (2005), three groups (6 carp in each group) were injected intraperitoneally (i.p.) with 50, 200, and 500 μg of MCLR/kg bw (low, medium, and high doses, respectively). No mortality was found during the experiment. In another group, 6 carp were injected i.p. with the same volume of 0.9 % saline solution as control. In the experiment, sampling time points were 3 and 24 h after MCLR injection. Three replicates of tissue were sampled from three different carp for each concentration and sampling time point, respectively. Approximately 100 mg of liver, kidney, intestine, brain, heart, muscle, spleen, and gill were excised, freed of attached tissue, and respectively stored in 1 ml of Trizol (Invitrogen) at −70 °C. Different tissues of 3 bighead carps without any treatment were also excised and used for tissues distribution analysis.

### RNA Extraction, Reverse Transcription, and RACE

Isolation, purification, and quantification of total RNA and first-strand cDNA synthesis were performed according to our previously described protocols (Li et al. 2009). Bighead carp *IL*-*8* cDNA fragments were initially isolated from an SSH cDNA library constructed with the mixed liver tissues of bighead carp at different time points after injection of 200 μg of MCLR/kg bw (unpublished data). Therefore, the hepatic RNA was used as template to amplify both ends of the *IL*-*8* cDNA. All primers used in this study are listed in Table 1. RACE was performed using SMART RACE cDNA Amplification kit (Clontech, Palo Alto, CA) according to the manufacturer’s instructions. Gene-specific primers of IL-8F1, IL-8F2, IL-8R1, and IL-8R2 were designed based on bighead carp *IL*-*8* cDNA fragments. For 5′ RACE, primers UPM, IL-8F1, and IL-8F2 were used, and the PCR conditions were as follows: 94 °C denaturation for 3 min, 30 cycles of 94 °C for 30 s, 60 °C for 30 s, 72 °C for 1 min, and 72 °C elongation for 7 min. For 3′ RACE, the cDNA template was transcribed by AMV Reverse Transcriptase (TaKaRa, Tokyo, Japan) with Oligo dT-Adaptor primer (Table 1). PCR was performed with the primers of 3′ Adaptor (Table 1), IL-8R1 and IL-8R2, and the PCR conditions were as follows: 94 °C denaturation for 2 min, 30 cycles of 94 °C for 30 s, 58 °C for 30 s, 72 °C for 1.5 min, and then 72 °C elongation for 7 min. The bands were extracted from agarose gel by using DNA Gel Extraction Kit.

**Table 1:** Primers used for cloning and expression studies

Primers	Sequence (5′–3′)	Application
IL-8F1	GTGAGGGCTGGGAGGGTAGA	5′ RACE & Q-PCR
IL-8F2	TGGTCAGAAATGCCACAGCAACA	5′ RACE
3′ CDS	AAGCAGTGGTATCAACGCAGAGTAC(T)_30_V N	3′ RACE
5′ CDS	(T)_25_V N	5′ RACE
IL-8R1	CCAAACTCCCTCATGAACTGCCTGCAGC	3′ RACE & Q-PCR
IL-8R2	CTCCACAGGGGGAAATGAAGTGCTCACA	3′ RACE
UPM	CTAATACGACTCACTATAGGGCAAGCAGTG GTATCAACGCAGAGT (long)	5′ RACE
	CTAATACGACTCACTATAGGGC (short)	
NUP	AAGCAGTGGTATCAACGCAGAGT	3′ RACE
IL-8F	TGTTGCTGTGGCATTTCTGACCA	Q-PCR & RT-PCR
IL-8R	GTGAGGGCTGGGAGGGTAGA	Q-PCR & RT-PCR
SMARTIIA oligo	AAGCAGTGGTATCAACGCAGAGTACGCGGG	5′ & 3′ RACE
GAPDH-F	GCCAGTCAGAACATTATCCCAGCCT	Internal control
GAPDH-R	GGTCCTCAGTGTATCCCAGAATGCC	Internal control

### TA Cloning, Sequencing, and Database Analysis

Purified PCR products were ligated into pMD18-T vectors (TaKaRa), and three plasmids were sequenced for each product, respectively. Sequences were analyzed based on nucleotide and protein databases using the BLAST program (http://www.ncbi.nlm.nih.gov/BLAST/). The protein prediction was performed using software at the ExPASy Molecular Biology Server (http://expasy.pku.edu.cn). The protein family signature was identified by InterPro (http://www.ebi.ac.uk/interpro/). The chemokine CXC domain was defined by National Center for Biotechnology Information (NCBI) conserved-domain database (http://www.ncbi.nlm.nih.gov/structure). Multiple sequence alignment was performed using the CLUSTALW 2 program (http://clustalw.genome.jp/). Phylogenetic tree was constructed by using the neighbor-joining (NJ) method with 1000 bootstrap in the Mega 5 software package.

### Q-PCR and Reverse Transcriptionase-PCR

Q-PCR was performed according to the method of Li et al. (2008) with Chromo 4 TM Continuous Fluorescence Detector from MJ Research using SYBR Green real-time PCR Master Mix (TOYOBO). Q-PCR conditions were as follows: 5 min at 95 °C, 40 cycles of 20 s at 94 °C, 20 s at 60 °C, and 20 s at 72 °C. Primers IL-8F and IL-8R were designed for quantitative analysis of *IL*-*8* mRNA in bighead carp (Table 1). Specificity of primers was confirmed by analyzing the melting curves and PCR product. Housekeeping gene glyceraldehyde 3-phosphate dehydrogenase (GAPDH) was used as internal standard. The plasmid that contained *IL*-*8* cDNA was used as positive control. Then standard curves were constructed with tenfold serially diluted plasmid. The *δC*_*t*_ method was used for quantification of target gene according to the manufacturer’s protocol (Applied Biosystems). The relative expression level (fold induction) of gene was presented as 2^(−ΔΔCT)^ method (Schmittgen and Zakrajsek 2000; Livak and Schmittgen 2001). All values are expressed as mean ± SD. One-way analysis of variance was used to elucidate if there are significant differences between the treatment and control groups (*P* < 0.05).

Reverse transcriptionase-PCR (RT-PCR) was performed according to the following protocol. Total RNA (2 μg) from various tissues of bighead carp in the untreated group was applied as template to synthesize the first strand of cDNA using AMV Reverse Transcriptase (TaKaRa, Japan) and oligo (dT)_18_ (TaKaRa, Japan). GAPDH was used as the internal standard. The cDNA was diluted and used as templates in PCR reactions with primers of IL-8F and IL-8R. The PCR program was as follows: one initial step of denaturation at 94 °C for 5 min, 30 cycles of 30 s at 94 °C, 30 s at 58 °C, 30 s at 72 °C, and a last extension step at 72 °C for 10 min. The amplified products were electrophoresed on agarose gels stained with ethidium bromide. Negative controls for each experimental group were performed by PCR without template.

## Results

### Sequence Analysis of Bighead Carp IL-8 cDNA

**Fig. 1:**
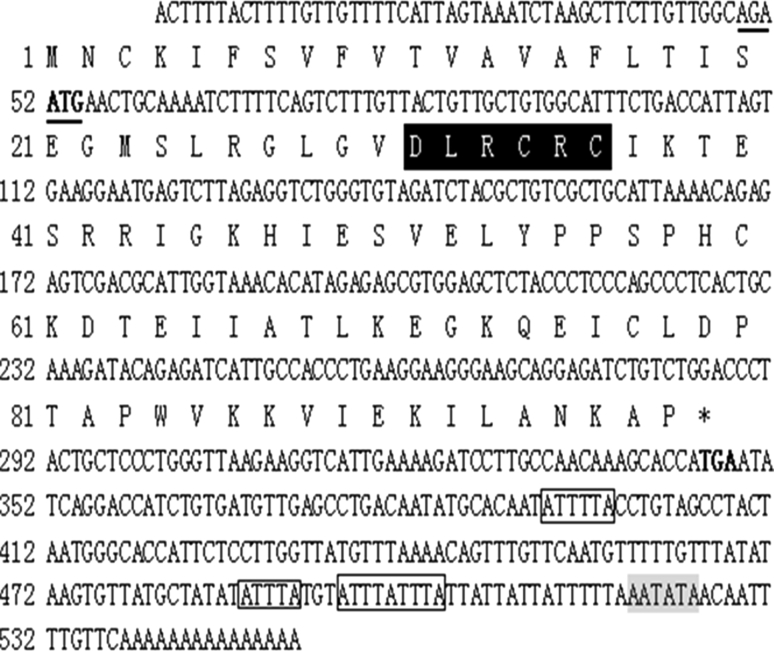
Full-length cDNA sequence of bighead carp *IL*-*8* and the deduced amino acids. The Kozak sequence is *underlined*. DLR motif and CXC motif are in *black* background. The AU-rich motifs are in *boxes*. The start and stop codons of the ORF are indicated in *bold*. The residue corresponding to the stop codon is noted by *asterisks*. The poly (A) signal sequence is *shaded*

The full-length cDNA of bighead carp IL-8 was 552 bp, containing a 51-bp 5′-UTR; a 204-bp 3′-UTR, including poly(A); and a 297-bp ORF. The deduced encoding product was a 98-amino acid polypeptide. Multiple instability motifs (1 ATTTA, 1 ATTTTA and 1 overlapping pentameric repeats ATTTATTTA) were present in the 3′-UTR of bighead carp *IL*-*8* cDNA. The poly(A) signal AATATA was 18 bp upstream of poly(A) (Fig. 1) (Tosi et al. 1981). The Kozak sequence -(A/G)NNATG-, recognized by ribosomes as the translational start site, was present within the 5′-UTR sequence. In addition, no ELR but DLR [aspartic acid (D)-leucine (L)-arginine (R)] motif was found in deduced bighead carp IL-8 protein, which was located immediately upstream of the CXC motif (CRC). Similar to that of common carp, there were also four cysteine residues (Cys 34, Cys 36, Cys 60, and Cys 77) in bighead carp IL-8.

The deduced IL-8 protein of bighead carp had a molecular weight of 10911.9 Da and an isoelectric point of 8.90. Based on the NCBI three-dimensional database, bighead carp IL-8 had a structure similar to that of human chemokine CXC (cd00273) containing typical functional parts such as tetramer interface, dimer interface, reporter binding cleft, receptor-binding site, glycosaminoglycan-binding site, N-loop, 30 s-loop, 40 s-loop, etc.

### IL-8 Sequence Similarity and Phylogenetic Analysis

**Fig. 2:**
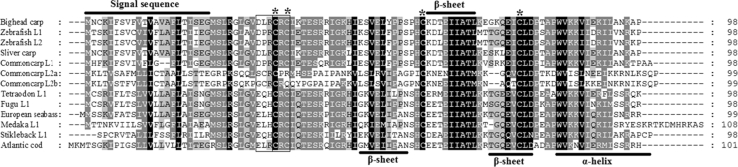
Alignment of the amino acid sequences of IL-8s from selected species. Correspondences of common names with Latin names and GenBank accession numbers are listed in Table 2. Residues with 100 % identity are shown in *dark gray boxes*, >80 % identity in *gray boxes*, and >60 % identity in *light gray boxes*. Residues with 100 % sequence identity are also displayed below the aligned sequences. The secondary structure elements from bighead carp IL-8 were shown with word annotation. The four cysteines are marked with *asterisks*

The deduced bighead IL-8 protein shared high identity with that of silver carp (97 %), common carp (82 %), and other teleostean (63–77 %) but low identity with mammalian IL-8 (Table 2). Multiple-sequence alignment was performed using the CLUSTALW 2 program (Fig. 2). Secondary structure predictions indicated that the bighead carp IL-8 molecule also contained three β-sheets and a carboxy-terminal α-helix (Fig. 2), similar to the structure of human IL-8 (cd00273). There was a signal sequence at the N-terminal.

**Table 2:** Amino acid identity comparing bighead carp IL-8 with other known IL-8s and chemokines

Species	Common name	Accession no.	Identity (%)	Query coverage (%)
*Hypophthalmichthys molitrix*	Silver carp	ACI02125.1	97.0	100.0
*Cyprinus carpio*	Common carp	ABE47600.1	82.0	100.0
*Oncorhynchus mykiss*	Rainbow trout	AAO25646.1	77.0	79.0
*Salmo trutta fario*	Brown trout	AAX45348.1	75.0	62.0
*S. salar*	Salmon	NP_001134182.1	65.0	97.0
*Dicentrarchus labrax*	European sea bass	CAM32186.1	65.0	97.0
*Gadus morhua*	Atlantic cod	CAD59734.2	63.0	80.0
*Takifugu rubripes*	Fugu rubripes	NP_001027759	60.2	75.5
*Xenopus tropicalis*	Xenopus	XP_002942577.1	55.0	78.0
*Meleagris gallopavo*	Turkey	XP_003205722.1	52.0	75.0
*Gallus gallus*	Chicken	NP_990349.1	51.0	75.0
*Melanogrammus aeglefinus*	Haddock	CAD97422	49.5	72.3
*Oreochromis niloticus*	Nile tilapia	ACU30057.1	49.5	68.7
*Anser cygnoides*	Chinese goose	BAE53442.1	47.0	95.0
*Pelodiscus sinensis*	Softshell turtle	ACP28489.1	45.0	94.0
*Columba livia*	Domestic pigeon	ABD49206	41.9	65.7
*Ornithorhynchus anatinus*	Platypus	XP_001511324	40.8	55.3
*Macaca mulatta*	Rhesus monkey	NP_001028137.1	39.0	55.2
*Homo sapiens*	Human	P10145.1	38.5	54.8
*Ovis aries*	Sheep	NP_001009401.1	37.1	52.4
*Canis lupus familiaris*	Dog	NP_001003200.1	34.0	50.5
*Mustela putorius furo*	Domestic ferret	BAF56573	33.0	52.4
*Bos taurus*	Cattle	NP_776350	33.0	50.9
*Sus scrofa*	Pig	NP_999032.1	32.7	49.0
*Equus caballus*	Horse	NP_001077420.1	29.1	49.5

**Fig. 3:**
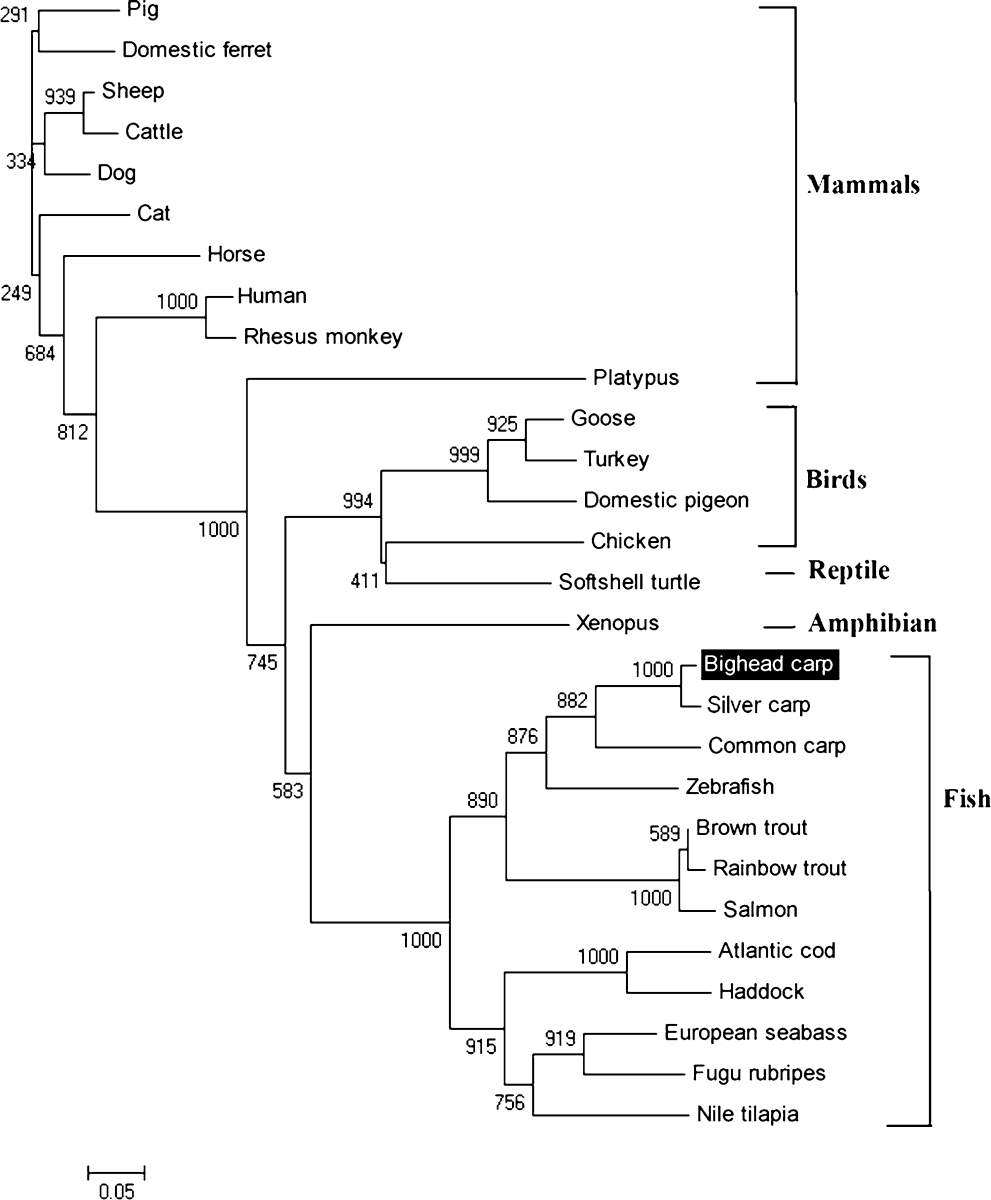
NJ tree of IL-8s from bighead carp and other species. Bootstrap values are indicated at the nodes. The evolutionary distance between two sequences was obtained by adding the lengths of the horizontal branches connecting them and using the *scale bar* (0.05 mutation/position)

The phylogenetic NJ-tree of vertebrate IL-8s was constructed based on the amino acid sequences listed in Table 2. As shown in Fig. 3, IL-8s of mammals, birds, and fish formed their own clades, respectively. Softshell turtle IL-8 was close to that of avian in evolution, and xenopus IL-8 was close to that of teleost. In clade of fish IL-8s, there were two clusters corresponding to two teleost IL-8 lineages, respectively. Teleost IL-8 lineage 1 included haddock, Atlantic cod, and other three fish species, whereas teleost IL-8 lineage 2 were mostly cyprinid-specific IL-8s, including carps, trouts, zebrafish, and salmon.

### Tissue Distribution of Bighead Carp IL-8 mRNA

**Fig. 4:**
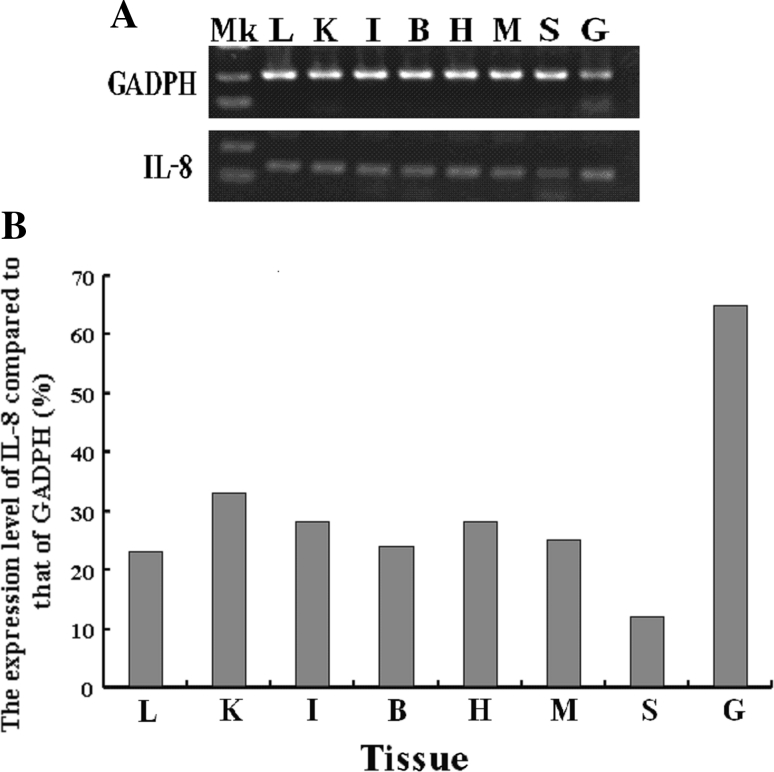
*IL*-*8* mRNA tissue distribution in bighead carp. Housekeeping gene *GAPDH* was used as internal control. **a** Results of RT-PCR. **b** Ratio of *IL*-*8* expression compared with GAPDH. *Mk* marker, *L* liver, *K* kidney, *I* intestine, *B* brain, *H* heart, *M* muscle, *S* spleen, *G* gill

The expression distribution of *IL*-*8* mRNA was examined in bighead carp using RT-PCR, and the result is shown in Fig. 4. It was found that under normal conditions, the transcription level of *IL*-*8* was low in liver, kidney, heart, intestine, gill, brain, and spleen. Compared to the expression level of *GAPDH* in each tissue, *IL*-*8* expression level was ≤35 % except in gill. No *IL*-*8* transcription was detected in negative control (data not shown).

### Expression Profiles of IL-8 in Bighead Carp Exposed to MCLR

**Fig. 5:**
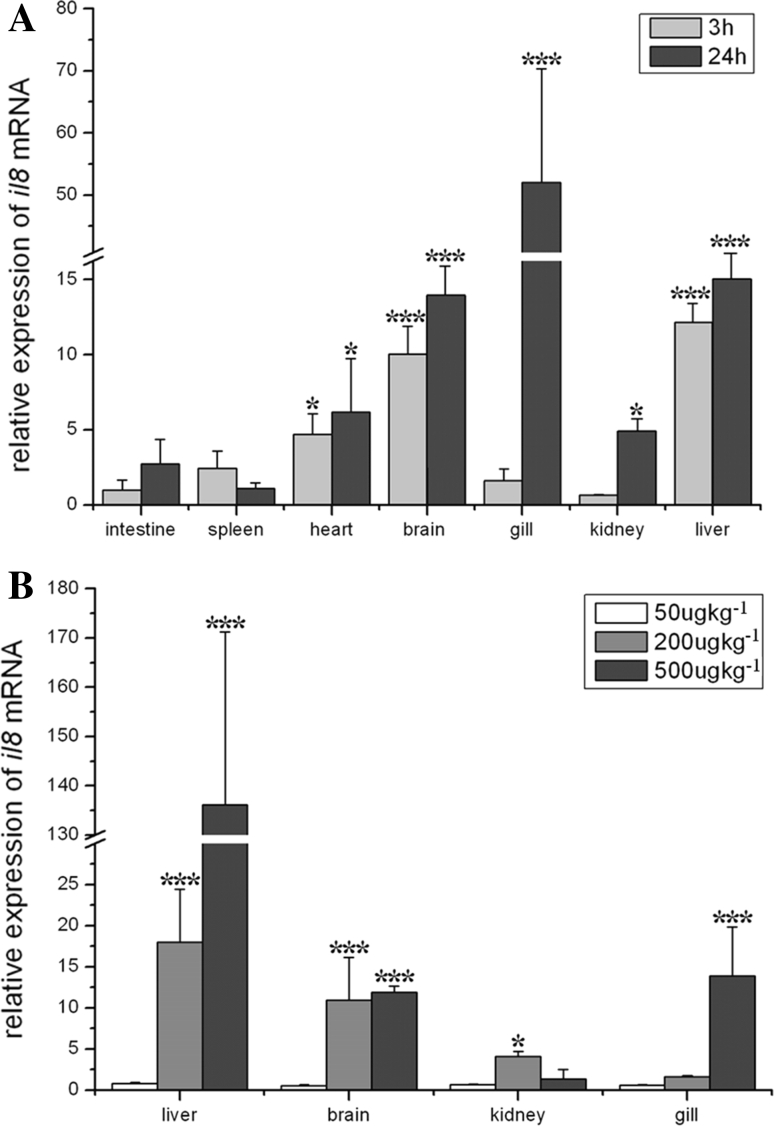
Expression profiles of *IL*-*8* in bighead carp injected with MCLR. Housekeeping gene *GAPDH* was used as internal control. Data are expressed as mean ± SD (*n* = 3). Significance levels observed were *p = 0.05 and ***p = 0.001. **a** *IL*-*8* expression profile in all tested tissues at 3 and 24 h after injection with MCLR 200 μg/kg bw (*n* = 3). **b** *IL*-*8* expression profile in liver, brain, kidney, and gill at 3 h after injection with 50, 200, and 500 μg/kg bw MCLR (*n* = 3)

The expression profiles of *IL*-*8* in bighead carp after MCLR injection are shown in Fig. 5. Housekeeping gene GAPDH was used as internal control. The expression profile of *IL*-*8* mRNA was quantified in various tissues of bighead carp injected with 200 μg of MCLR/kg bw at 3 and 24 h after exposure (Fig. 5a). In intestine and spleen, expression of *IL*-*8* was not changed by injection of MCLR compared with controls. In heart, transcription of *IL*-*8* was upregulated significantly by fivefold at 3 h and by sixfold at 24 h compared with controls. In brain and liver, expression of *IL*-*8* was increased to >tenfold at 3 h and then increased to >14-fold at 24 h after injection. In gill, *IL*-*8* transcription was at a normal level at 3 h; however, it increased to ~50-fold at 24 h compared with controls. Just like in gill, expression of *IL*-*8* mRNA was at a normal level in kidney at 3 h; however, it was significantly increased to fivefold at 24 h after injection compared with controls.

The expression profile of *IL*-*8* in bighead carp after 3 h of injection with different doses of MCLR is shown in Fig. 5b. In all tested tissues, expression of *IL*-*8* was not changed by the low dose of MCLR (50 µg/kg bw [low-dose group]). Under medium and high doses of MCLR (200 and 500 µg/kg bw [medium- and high-dose groups, respectively]), however, expression of *IL*-*8* was generally upregulated compared with controls. Especially in liver, transcription of *IL*-*8* was significantly increased to 18-fold in the medium-dose group and 135-fold in the high-dose group, respectively, compared with controls. In brain, *IL*-*8* expression increased to >tenfold in the medium- and high-dose groups compared with controls. In kidney, *IL*-*8* transcription was significantly upregulated only in the medium-dose group, whereas in gill it was significantly induced only in the high-dose group. Except for kidney, MCLR induced *IL*-*8* expression in a dose-dependent pattern.

## Discussion

In the present study, we cloned the full-length cDNA of *IL*-*8* in bighead carp for the first time. Inflammatory genes often contain the adenylate-uridylate (AU)-rich sequence at 3′-UTR, which help to decrease RNA stability. Therefore, their transcripts are usually unstable and have transient expression (Caput et al. 1986). In this study, multiple AU-rich motifs were also present in bighead carp *IL*-*8* cDNA, suggesting that its transcript is unstable in vivo, which was in accordance with the previous description on fish chemokines (Roca et al. 2007). In addition, some other typical features were also founded in deduced bighead carp IL-8 protein, including the signal peptide, a DLR motif, four cysteine residues, and a CXC domain. Four cysteine residues were essential for the formation of the tertiary structure and consequently the function of CXC chemokines (Rajarathnam et al. 1999). The DLR motif in bighead carp IL-8 neighbored the CRC residues, and this is consistent with those of other fish species (Abdelkhalek et al. 2009). The CXC chemokines without ELR motif are considered to mainly attract lymphocytes and monocytes in humans (Laing and Secombes 2004). Therefore, the function analysis of bighead carp IL-8 protein in response to MCs might be of great significance.

In this study, the deduced bighead carp IL-8 protein shared high identities with that of other species, indicating that IL-8 was conserved in evolution. However, it shared low identities with mammalian IL-8s. It was known that there was only one conserved IL-8 lineage in mammals, whereas there are at least two IL-8 lineages in teleosts. Bighead carp IL-8 was classified into teleost IL-8 lineage 2, which was not the direct orthologue of mammalian IL-8. It is interesting that both lineages of carp IL-8s appeared to be functional homologs of mammalian IL8, but their different induction requirements and kinetics evoked a gene-specific subfunctionalization (Van der Aa et al. 2010). In present study, the fish clade of IL-8s could be separated into two distinct subgroups, which was in accordance with previous reports that there are two IL-8 lineages in fish (Abdelkhalek et al. 2009). The two IL-8-like lineages might have diverged through a chromosome duplication that was believed to have occurred in a teleost ancestor (Ravi and Venkatesh 2008). The mammalian IL-8 clade is not close to the fish IL-8 clade. Because IL-8 proteins are short and evolve quickly, it was often difficult to show more than a tendency in distance by phylogenetic analysis, especially when only a few sequences were available (Van der Aa et al. 2010).

Based on RT-PCR analysis, bighead carp *IL*-*8* gene was expressed constitutively in various tissues, which was consistent with the report in common carp and other species (Corripio-Miyar et al. 2007; Seppola et al. 2008; Van der Aa et al. 2010). The expression of *IL*-*8* was relatively higher in gill than in other tested tissues. This might be a result of the function of gill. In fish, material exchange between inside and outside environments are mainly implemented through gill. Therefore, more IL-8 and other signal molecules might be necessary in the host defense system for promoting migration of neutrophils to inflammatory sites and their subsequent adherence to endothelial cells (Hoffmann et al. 2002).

*IL*-*8* gene was an inducible acute-phase inflammatory gene, and its expression was sensitive to oxidative stress (DeForge et al. 1993). MCLR toxicity included activity inhibition of PP2A and PP1, as well as induction of oxidative stress, leading to liver damage and liver tumor promotion (Butler et al. 2009). Moreover, prolonged exposure to MCLR also induces inflammatory reactions (Guzman and Solter 1999). The oxidative stress induced by MCLR occurred in a time- and dose-dependent pattern (Zhang et al. 2009). In this study, Q-PCR analysis showed that the expression of *IL*-*8* mRNA was also increased in a time- and dose-dependent pattern in bighead carp injected with MCLR. Further study is necessary to investigate whether there is a relationship between the upregulation of *IL*-*8* and MCLR-induced oxidative stress or MCLR-stimulated acute inflammation in bighead carp.

In this study, dramatically increased expression of *IL*-*8* was found in liver of bighead carp exposed to MCLR, even reaching >100-fold in the high-dose group compared with controls. The dynamic change in expression level was a typical property of *IL*-*8* gene. In healthy tissues, the expression level of *IL*-*8* was low, but it could be rapidly induced by 10- to 100-fold by various elicitors, such as TNF-α and IL-1β, bacteria, viral products, or cellular stresses (Abdelkhalek et al. 2009). Evidences have confirmed that overexpression of *IL*-*8* is related to liver disease (Zimmermann et al. 2011). *IL*-*8* expression was found to be upregulated in the circulation and the liver of chronic liver disease patients, and activation of *IL*-*8* was linked to progression of liver fibrosis. Therefore, our result might also provide some clue to explain why the most common symptom of poisoning in humans and other animals with MCs was liver damage (Butler et al. 2009).

## Conclusion

We cloned the full-length cDNA of *IL*-*8* in bighead carp for the first time. The deduced IL-8 protein contained both a DLR and a CXC motif and shared high identity with IL-8s from other fish species. Phylogenetic analysis showed that bighead carp IL-8 protein was classified into the teleost lineage 2. The expression of bighead carp *IL*-*8* gene could be significantly upregulated by MCLR exposure in a temporal- and dose-dependent pattern. The present study was useful for understanding anti-MCLR immunity in bighead carp.
